# Bis{4-chloro-2-[(2-hy­droxy­eth­yl)imino­meth­yl]phenolato}nickel(II) monohydrate

**DOI:** 10.1107/S1600536811031680

**Published:** 2011-08-11

**Authors:** Chen-Yi Wang, Jin-Yun Ye, Xiang Wu, Zhi-Ping Han

**Affiliations:** a Department of Chemistry, Huzhou University, Huzhou 313000, People’s Republic of China

## Abstract

The title mononuclear nickel(II) complex, [Ni(C_9_H_9_ClNO_2_)_2_]·H_2_O, was obtained by the reaction of 5-chloro­salicyl­aldehyde, 2-amino­ethanol and nickel nitrate in methanol. The Ni atom is six-coordinated by two phenolate O, two imine N and two hy­droxy O atoms from two crystallographically different Schiff base ligands, forming an octa­hedral geometry. In the crystal, mol­ecules are linked through inter­molecular O—H⋯O and O—H⋯Cl hydrogen bonds.

## Related literature

For our investigations of urease inhibitors, see: Wang (2009 ▶); Wang & Ye (2011 ▶). For similar nickel(II) complexes, see: Arıcı *et al.* (2005 ▶); Liu *et al.* (2006 ▶); Li & Wang (2007 ▶); Ali *et al.* (2006 ▶).
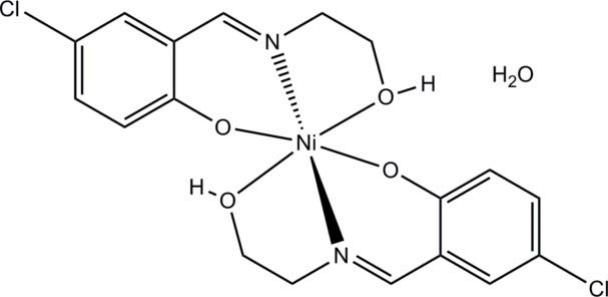


## Experimental

### 

#### Crystal data

[Ni(C_9_H_9_ClNO_2_)_2_]·H_2_O
*M* *_r_* = 473.97
Orthorhombic, 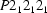

*a* = 9.846 (1) Å
*b* = 12.646 (2) Å
*c* = 16.006 (2) Å
*V* = 1992.9 (4) Å^3^
*Z* = 4
Mo *K*α radiation
μ = 1.27 mm^−1^
*T* = 298 K
0.30 × 0.27 × 0.27 mm

#### Data collection

Bruker SMART CCD area-detector diffractometer
Absorption correction: multi-scan (*SADABS*; Sheldrick, 1996 ▶) *T*_min_ = 0.701, *T*_max_ = 0.725
11691 measured reflections
4328 independent reflections
3147 reflections with *I* > 2σ(*I*)
*R*_int_ = 0.048

#### Refinement

*R*[*F* ^2^ > 2σ(*F* ^2^)] = 0.039
*wR*(*F* ^2^) = 0.076
*S* = 1.04
4328 reflections
265 parameters
5 restraints
H atoms treated by a mixture of independent and constrained refinement
Δρ_max_ = 0.35 e Å^−3^
Δρ_min_ = −0.39 e Å^−3^
Absolute structure: Flack (1983 ▶), 1855 Friedel pairs
Flack parameter: 0.015 (15)

### 

Data collection: *SMART* (Bruker, 1998 ▶); cell refinement: *SAINT* (Bruker, 1998 ▶); data reduction: *SAINT*; program(s) used to solve structure: *SHELXS97* (Sheldrick, 2008 ▶); program(s) used to refine structure: *SHELXL97* (Sheldrick, 2008 ▶); molecular graphics: *SHELXTL* (Sheldrick, 2008 ▶); software used to prepare material for publication: *SHELXL97*.

## Supplementary Material

Crystal structure: contains datablock(s) global, I. DOI: 10.1107/S1600536811031680/om2456sup1.cif

Structure factors: contains datablock(s) I. DOI: 10.1107/S1600536811031680/om2456Isup2.hkl

Additional supplementary materials:  crystallographic information; 3D view; checkCIF report

## Figures and Tables

**Table 1:** Selected bond lengths (Å)

Ni1—N2	1.996 (3)
Ni1—N1	2.000 (3)
Ni1—O3	2.011 (2)
Ni1—O1	2.015 (2)
Ni1—O2	2.131 (2)
Ni1—O4	2.160 (3)

**Table 2:** Hydrogen-bond geometry (Å, °)

*D*—H⋯*A*	*D*—H	H⋯*A*	*D*⋯*A*	*D*—H⋯*A*
O5—H5*B*⋯O1^i^	0.86 (1)	2.00 (2)	2.846 (4)	167 (4)
O4—H4⋯O5^ii^	0.85 (1)	1.97 (2)	2.798 (4)	165 (4)
O2—H2⋯O3^iii^	0.85 (1)	1.87 (2)	2.699 (3)	165 (4)
O5—H5*A*⋯Cl2	0.84 (1)	2.73 (2)	3.542 (4)	163 (4)

Symmetry codes: (i) 
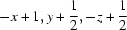
; (ii) 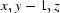; (iii) 
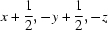
.

**Fig. 1.**
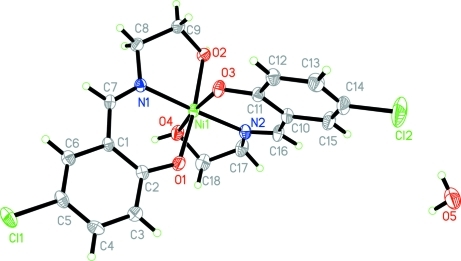
The molecular structure of the title compound, showing the atom-numbering scheme. Displacement ellipsoids are drawn at the 30% probability level.

**Fig. 2.**
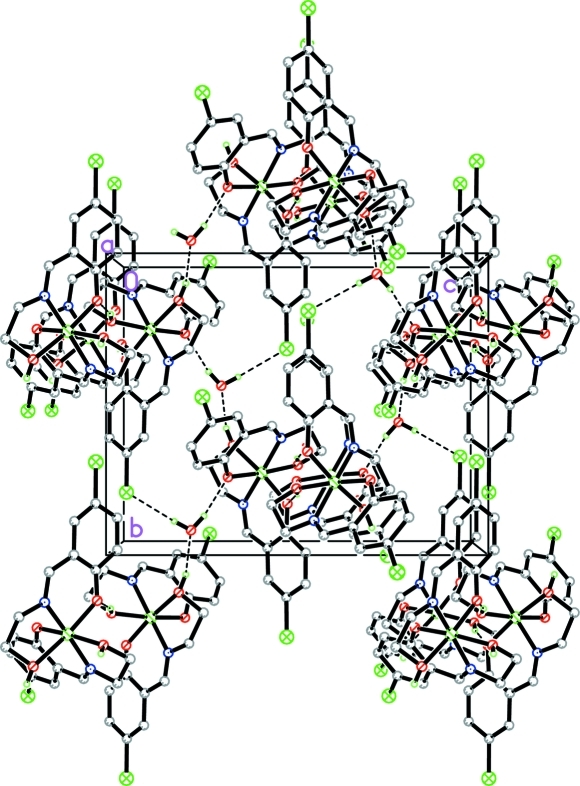
The molecular packing of the title compound, viewed along the a axis. Intermolecular hydrogen bonds are shown as dashed lines.

## supplementary crystallographic information

### Comment

As part of our investigations into novel urease inhibitors (Wang & Ye, 2011; Wang, 2009), we have synthesized the title compound, a new mononuclear nickel(II) complex, Fig. 1. The compound contains a mononuclear nickel(II) complex molecule and a water molecule of crystallization. The Ni atom in the complex is six-coordinated by two phenolate O, two imine N, and two hydroxy O atoms from two Schiff base ligands, forming an octahedral geometry. The *trans* angles at the Ni atom are in the range 172.5 (1)–174.1 (1)°; the other angles are close to 90°, ranging from 80.1 (1) to 94.9 (1)°, indicating a slightly distorted octahedral coordination. The Ni–O and Ni–N bond lengths (Table 1) are typical and are comparable with those observed in other similar nickel(II) complexes (Arıcı *et al.*, 2005; Liu *et al.*, 2006; Li & Wang, 2007; Ali *et al.*, 2006).

In the crystal structure of the compound, molecules are linked through intermolecular O—H···O and O—H···Cl hydrogen bonds (Table 2), to form a three-dimensional network (Fig. 2).

### Experimental

5-Chlorosalicylaldehyde (1.0 mmol, 0.157 g), 2-aminoethanol (1.0 mmol, 0.061 g), and nickel nitrate hexahydrate (0.5 mmol, 0.146 g) were dissolved in MeOH (30 ml). The mixture was stirred at room temperature for 10 min to give a clear green solution. After keeping the solution in air for a week, green block-shaped crystals were formed at the bottom of the vessel.

### Refinement

The water and hydroxy H atoms were located from a difference Fourier map and refined isotropically, with O—H and H···H distances restrained to 0.85 (1) and 1.37 (2) Å, respectively. Their isotropic displacement parameters were fixed at 0.08 Å^2^). The remaining H atoms were placed in geometrically idealized positions and constrained to ride on their parent atoms, with C—H distances in the range 0.93–0.97 Å, and with *U*_iso_(H) set at 1.2*U*_eq_(C and O).

### Crystal data

**Table:**

[Ni(C_9_H_9_ClNO_2_)_2_]·H_2_O	*D*_x_ = 1.580 Mg m^−^^3^
*M**_r_* = 473.97	Mo *K*α radiation, λ = 0.71073 Å
Orthorhombic, *P*2_1_2_1_2_1_	Cell parameters from 2792 reflections
*a* = 9.846 (1) Å	θ = 2.4–24.5°
*b* = 12.646 (2) Å	µ = 1.27 mm^−^^1^
*c* = 16.006 (2) Å	*T* = 298 K
*V* = 1992.9 (4) Å^3^	Block, green
*Z* = 4	0.30 × 0.27 × 0.27 mm
*F*(000) = 976	

### Data collection

**Table:**

Bruker SMART CCD area-detector diffractometer	4328 independent reflections
Radiation source: fine-focus sealed tube	3147 reflections with *I* > 2σ(*I*)
graphite	*R*_int_ = 0.048
ω scans	θ_max_ = 27.0°, θ_min_ = 2.4°
Absorption correction: multi-scan (*SADABS*; Sheldrick, 1996)	*h* = −12→12
*T*_min_ = 0.701, *T*_max_ = 0.725	*k* = −14→16
11691 measured reflections	*l* = −20→14

### Refinement

**Table:**

Refinement on *F*^2^	Secondary atom site location: difference Fourier map
Least-squares matrix: full	Hydrogen site location: inferred from neighbouring sites
*R*[*F*^2^ > 2σ(*F*^2^)] = 0.039	H atoms treated by a mixture of independent and constrained refinement
*wR*(*F*^2^) = 0.076	*w* = 1/[σ^2^(*F*_o_^2^) + (0.0206*P*)^2^ + 0.1322*P*] where *P* = (*F*_o_^2^ + 2*F*_c_^2^)/3
*S* = 1.04	(Δ/σ)_max_ < 0.001
4328 reflections	Δρ_max_ = 0.35 e Å^−^^3^
265 parameters	Δρ_min_ = −0.39 e Å^−^^3^
5 restraints	Absolute structure: Flack (1983), 1855 Friedel pairs
Primary atom site location: structure-invariant direct methods	Flack parameter: 0.015 (15)

### Special details

**Table:**

Geometry. All e.s.d.'s (except the e.s.d. in the dihedral angle between two l.s. planes) are estimated using the full covariance matrix. The cell e.s.d.'s are taken into account individually in the estimation of e.s.d.'s in distances, angles and torsion angles; correlations between e.s.d.'s in cell parameters are only used when they are defined by crystal symmetry. An approximate (isotropic) treatment of cell e.s.d.'s is used for estimating e.s.d.'s involving l.s. planes.
Refinement. Refinement of *F*^2^ against ALL reflections. The weighted *R*-factor *wR* and goodness of fit *S* are based on *F*^2^, conventional *R*-factors *R* are based on *F*, with *F* set to zero for negative *F*^2^. The threshold expression of *F*^2^ > σ(*F*^2^) is used only for calculating *R*-factors(gt) *etc*. and is not relevant to the choice of reflections for refinement. *R*-factors based on *F*^2^ are statistically about twice as large as those based on *F*, and *R*- factors based on ALL data will be even larger.

### Fractional atomic coordinates and isotropic or equivalent isotropic displacement parameters (Å^2^)

**Table:**

	*x*	*y*	*z*	*U*_iso_*/*U*_eq_	
Ni1	0.53364 (4)	0.24034 (3)	0.09823 (3)	0.03031 (12)	
Cl1	−0.07950 (10)	−0.02347 (9)	0.24602 (7)	0.0569 (3)	
Cl2	0.4617 (2)	0.80769 (9)	0.02875 (10)	0.1053 (6)	
N1	0.4422 (3)	0.1170 (2)	0.04386 (17)	0.0293 (7)	
N2	0.6463 (3)	0.3534 (2)	0.15079 (19)	0.0331 (7)	
O1	0.3912 (2)	0.2511 (2)	0.18821 (14)	0.0396 (6)	
O2	0.6727 (2)	0.21542 (19)	−0.00183 (15)	0.0351 (6)	
H2	0.7545 (17)	0.195 (3)	0.000 (3)	0.080*	
O3	0.4326 (2)	0.34479 (18)	0.02676 (15)	0.0353 (6)	
O4	0.6595 (3)	0.1451 (2)	0.17926 (17)	0.0427 (7)	
H4	0.634 (4)	0.0850 (18)	0.198 (3)	0.080*	
O5	0.5861 (4)	0.9351 (2)	0.2104 (2)	0.0666 (9)	
H5A	0.575 (5)	0.905 (3)	0.1640 (12)	0.080*	
H5B	0.601 (4)	0.886 (2)	0.2461 (17)	0.080*	
C1	0.2544 (3)	0.1029 (3)	0.1421 (2)	0.0285 (8)	
C2	0.2870 (3)	0.1881 (3)	0.1969 (2)	0.0312 (9)	
C3	0.1979 (3)	0.2045 (3)	0.2648 (2)	0.0369 (9)	
H3	0.2154	0.2602	0.3012	0.044*	
C4	0.0863 (4)	0.1416 (3)	0.2794 (2)	0.0385 (9)	
H4A	0.0302	0.1547	0.3250	0.046*	
C5	0.0581 (3)	0.0591 (3)	0.2259 (2)	0.0377 (10)	
C6	0.1391 (3)	0.0405 (3)	0.1584 (2)	0.0353 (9)	
H6	0.1177	−0.0147	0.1223	0.042*	
C7	0.3306 (3)	0.0749 (3)	0.0684 (2)	0.0317 (9)	
H7	0.2953	0.0210	0.0354	0.038*	
C8	0.5114 (3)	0.0774 (3)	−0.0304 (2)	0.0376 (9)	
H8A	0.4449	0.0547	−0.0714	0.045*	
H8B	0.5671	0.0170	−0.0157	0.045*	
C9	0.6000 (4)	0.1642 (3)	−0.0674 (2)	0.0409 (10)	
H9A	0.6634	0.1339	−0.1071	0.049*	
H9B	0.5438	0.2152	−0.0966	0.049*	
C10	0.5384 (4)	0.5021 (3)	0.0821 (2)	0.0361 (9)	
C11	0.4462 (3)	0.4476 (3)	0.0297 (2)	0.0322 (9)	
C12	0.3640 (4)	0.5102 (3)	−0.0228 (2)	0.0398 (10)	
H12	0.3043	0.4765	−0.0592	0.048*	
C13	0.3680 (4)	0.6183 (3)	−0.0226 (3)	0.0486 (11)	
H13	0.3114	0.6569	−0.0578	0.058*	
C14	0.4562 (6)	0.6693 (3)	0.0297 (3)	0.0550 (12)	
C15	0.5406 (4)	0.6136 (3)	0.0800 (2)	0.0512 (11)	
H15	0.6014	0.6498	0.1141	0.061*	
C16	0.6327 (4)	0.4522 (3)	0.1399 (2)	0.0395 (10)	
H16	0.6877	0.4966	0.1715	0.047*	
C17	0.7482 (4)	0.3129 (3)	0.2095 (3)	0.0476 (12)	
H17A	0.7642	0.3645	0.2532	0.057*	
H17B	0.8331	0.3006	0.1804	0.057*	
C18	0.6985 (4)	0.2117 (3)	0.2472 (3)	0.0497 (11)	
H18A	0.7699	0.1785	0.2797	0.060*	
H18B	0.6215	0.2250	0.2835	0.060*	

### Atomic displacement parameters (Å^2^)

**Table:**

	*U*^11^	*U*^22^	*U*^33^	*U*^12^	*U*^13^	*U*^23^
Ni1	0.02362 (18)	0.0263 (2)	0.0410 (3)	−0.0021 (2)	−0.0013 (2)	−0.0026 (2)
Cl1	0.0378 (6)	0.0680 (7)	0.0648 (8)	−0.0153 (5)	0.0081 (6)	0.0168 (6)
Cl2	0.1734 (16)	0.0291 (6)	0.1135 (12)	−0.0054 (9)	−0.0607 (13)	0.0109 (6)
N1	0.0269 (17)	0.0244 (15)	0.0367 (18)	0.0013 (13)	0.0017 (14)	−0.0003 (14)
N2	0.0255 (16)	0.0317 (18)	0.042 (2)	−0.0035 (14)	−0.0052 (14)	0.0019 (15)
O1	0.0363 (12)	0.0363 (14)	0.0463 (15)	−0.0070 (13)	0.0054 (11)	−0.0114 (15)
O2	0.0233 (11)	0.0422 (16)	0.0398 (15)	−0.0034 (11)	0.0018 (12)	−0.0067 (12)
O3	0.0255 (14)	0.0290 (13)	0.0513 (17)	−0.0018 (11)	−0.0073 (12)	−0.0043 (12)
O4	0.0506 (18)	0.0326 (15)	0.0449 (18)	−0.0009 (14)	−0.0107 (15)	0.0006 (14)
O5	0.079 (2)	0.0501 (19)	0.071 (2)	−0.0112 (18)	−0.004 (2)	0.0169 (16)
C1	0.0223 (17)	0.028 (2)	0.035 (2)	−0.0022 (15)	−0.0032 (16)	0.0050 (16)
C2	0.0274 (19)	0.030 (2)	0.036 (2)	−0.0001 (16)	−0.0022 (17)	0.0039 (17)
C3	0.0323 (19)	0.038 (2)	0.041 (3)	0.0041 (16)	−0.0019 (18)	−0.0024 (18)
C4	0.0312 (19)	0.049 (2)	0.035 (2)	0.0061 (19)	0.0062 (17)	0.008 (2)
C5	0.024 (2)	0.042 (2)	0.047 (3)	−0.0032 (17)	−0.0015 (18)	0.0138 (19)
C6	0.0302 (19)	0.038 (2)	0.037 (2)	−0.0020 (17)	−0.0038 (18)	0.0033 (19)
C7	0.0293 (19)	0.0265 (19)	0.039 (2)	−0.0040 (16)	−0.0037 (17)	0.0002 (16)
C8	0.033 (2)	0.041 (2)	0.039 (2)	−0.0057 (17)	0.0062 (17)	−0.0090 (17)
C9	0.036 (2)	0.047 (2)	0.039 (2)	−0.0118 (18)	0.0011 (18)	−0.0060 (19)
C10	0.0386 (19)	0.0278 (18)	0.042 (2)	−0.0028 (18)	−0.003 (2)	0.0008 (16)
C11	0.026 (2)	0.032 (2)	0.039 (2)	−0.0033 (16)	0.0021 (17)	−0.0031 (17)
C12	0.036 (2)	0.037 (2)	0.046 (3)	−0.0033 (18)	−0.0059 (18)	0.003 (2)
C13	0.057 (3)	0.040 (2)	0.049 (3)	0.005 (2)	−0.010 (2)	0.010 (2)
C14	0.086 (3)	0.026 (2)	0.053 (3)	−0.003 (2)	−0.006 (3)	0.0055 (19)
C15	0.068 (3)	0.033 (2)	0.053 (3)	−0.009 (2)	−0.016 (3)	−0.0025 (19)
C16	0.034 (2)	0.031 (2)	0.054 (3)	−0.0088 (17)	−0.0082 (18)	−0.0051 (19)
C17	0.039 (2)	0.043 (2)	0.060 (3)	−0.006 (2)	−0.021 (2)	−0.005 (2)
C18	0.052 (2)	0.043 (3)	0.055 (3)	0.0039 (19)	−0.019 (2)	0.002 (2)

### Geometric parameters (Å, °)

**Table:**

Ni1—N2	1.996 (3)	C4—C5	1.377 (5)
Ni1—N1	2.000 (3)	C4—H4A	0.9300
Ni1—O3	2.011 (2)	C5—C6	1.363 (5)
Ni1—O1	2.015 (2)	C6—H6	0.9300
Ni1—O2	2.131 (2)	C7—H7	0.9300
Ni1—O4	2.160 (3)	C8—C9	1.522 (5)
Cl1—C5	1.741 (3)	C8—H8A	0.9700
Cl2—C14	1.751 (4)	C8—H8B	0.9700
N1—C7	1.283 (4)	C9—H9A	0.9700
N1—C8	1.458 (4)	C9—H9B	0.9700
N2—C16	1.269 (4)	C10—C15	1.410 (5)
N2—C17	1.467 (4)	C10—C11	1.415 (5)
O1—C2	1.307 (4)	C10—C16	1.454 (5)
O2—C9	1.425 (4)	C11—C12	1.410 (5)
O2—H2	0.847 (10)	C12—C13	1.368 (5)
O3—C11	1.308 (4)	C12—H12	0.9300
O4—C18	1.428 (4)	C13—C14	1.367 (5)
O4—H4	0.852 (10)	C13—H13	0.9300
O5—H5B	0.860 (10)	C14—C15	1.355 (5)
O5—H5A	0.844 (10)	C15—H15	0.9300
C1—C6	1.407 (4)	C16—H16	0.9300
C1—C2	1.426 (5)	C17—C18	1.497 (5)
C1—C7	1.442 (5)	C17—H17A	0.9700
C2—C3	1.412 (5)	C17—H17B	0.9700
C3—C4	1.377 (5)	C18—H18A	0.9700
C3—H3	0.9300	C18—H18B	0.9700
			
N2—Ni1—N1	172.89 (12)	N1—C7—C1	126.4 (3)
N2—Ni1—O3	92.54 (11)	N1—C7—H7	116.8
N1—Ni1—O3	92.40 (10)	C1—C7—H7	116.8
N2—Ni1—O1	92.13 (11)	N1—C8—C9	109.7 (3)
N1—Ni1—O1	92.89 (10)	N1—C8—H8A	109.7
O3—Ni1—O1	91.03 (10)	C9—C8—H8A	109.7
N2—Ni1—O2	93.76 (11)	N1—C8—H8B	109.7
N1—Ni1—O2	81.20 (10)	C9—C8—H8B	109.7
O3—Ni1—O2	89.28 (10)	H8A—C8—H8B	108.2
O1—Ni1—O2	174.08 (10)	O2—C9—C8	109.2 (3)
N2—Ni1—O4	80.06 (11)	O2—C9—H9A	109.8
N1—Ni1—O4	94.87 (11)	C8—C9—H9A	109.8
O3—Ni1—O4	172.50 (10)	O2—C9—H9B	109.8
O1—Ni1—O4	90.43 (10)	C8—C9—H9B	109.8
O2—Ni1—O4	90.02 (10)	H9A—C9—H9B	108.3
C7—N1—C8	120.4 (3)	C15—C10—C11	118.9 (3)
C7—N1—Ni1	125.2 (3)	C15—C10—C16	116.0 (3)
C8—N1—Ni1	114.3 (2)	C11—C10—C16	125.1 (3)
C16—N2—C17	120.3 (3)	O3—C11—C12	118.5 (3)
C16—N2—Ni1	126.0 (3)	O3—C11—C10	124.8 (3)
C17—N2—Ni1	113.6 (2)	C12—C11—C10	116.7 (3)
C2—O1—Ni1	125.6 (2)	C13—C12—C11	122.9 (4)
C9—O2—Ni1	107.31 (19)	C13—C12—H12	118.6
C9—O2—H2	111 (3)	C11—C12—H12	118.6
Ni1—O2—H2	129 (3)	C14—C13—C12	119.4 (4)
C11—O3—Ni1	125.6 (2)	C14—C13—H13	120.3
C18—O4—Ni1	106.4 (2)	C12—C13—H13	120.3
C18—O4—H4	110 (3)	C15—C14—C13	120.6 (4)
Ni1—O4—H4	123 (3)	C15—C14—Cl2	120.4 (4)
H5B—O5—H5A	106 (2)	C13—C14—Cl2	119.0 (4)
C6—C1—C2	119.4 (3)	C14—C15—C10	121.6 (4)
C6—C1—C7	115.7 (3)	C14—C15—H15	119.2
C2—C1—C7	124.9 (3)	C10—C15—H15	119.2
O1—C2—C3	118.7 (3)	N2—C16—C10	125.6 (3)
O1—C2—C1	124.9 (3)	N2—C16—H16	117.2
C3—C2—C1	116.4 (3)	C10—C16—H16	117.2
C4—C3—C2	122.8 (3)	N2—C17—C18	109.5 (3)
C4—C3—H3	118.6	N2—C17—H17A	109.8
C2—C3—H3	118.6	C18—C17—H17A	109.8
C3—C4—C5	119.5 (4)	N2—C17—H17B	109.8
C3—C4—H4A	120.2	C18—C17—H17B	109.8
C5—C4—H4A	120.2	H17A—C17—H17B	108.2
C6—C5—C4	120.4 (3)	O4—C18—C17	106.6 (3)
C6—C5—Cl1	119.9 (3)	O4—C18—H18A	110.4
C4—C5—Cl1	119.8 (3)	C17—C18—H18A	110.4
C5—C6—C1	121.5 (3)	O4—C18—H18B	110.4
C5—C6—H6	119.3	C17—C18—H18B	110.4
C1—C6—H6	119.3	H18A—C18—H18B	108.6

### Hydrogen-bond geometry (Å, °)

**Table:**

*D*—H···*A*	*D*—H	H···*A*	*D*···*A*	*D*—H···*A*
O5—H5B···O1^i^	0.86 (1)	2.00 (2)	2.846 (4)	167 (4)
O4—H4···O5^ii^	0.85 (1)	1.97 (2)	2.798 (4)	165 (4)
O2—H2···O3^iii^	0.85 (1)	1.87 (2)	2.699 (3)	165 (4)
O5—H5A···Cl2	0.84 (1)	2.73 (2)	3.542 (4)	163 (4)

Symmetry codes: (i) −*x*+1, *y*+1/2, −*z*+1/2; (ii) *x*, *y*−1, *z*; (iii) *x*+1/2, −*y*+1/2, −*z*.
